# Complete mitochondrial genome of the aluminum-tolerant fungus *Rhodotorula taiwanensis* RS1 and comparative analysis of *Basidiomycota* mitochondrial genomes

**DOI:** 10.1002/mbo3.74

**Published:** 2013-02-21

**Authors:** Xue Qiang Zhao, Tomoko Aizawa, Jessica Schneider, Chao Wang, Ren Fang Shen, Michio Sunairi

**Affiliations:** 1 Department of Applied Biological Sciences, College of Bioresouce Sciences, Nihon University1866 Kameino, Fujisawa, Kanagawa, Japan; 2 State Key Laboratory of Soil and Sustainable Agriculture, Institute of Soil Science, Chinese Academy of SciencesNanjing, China; 3 Computational Genomics, Center for Biotechnology, Bielefeld UniversityBielefeld, Germany

**Keywords:** Annotation, basidiomycetous yeasts, comparative genomics

## Abstract

The complete mitochondrial genome of *Rhodotorula taiwanensis* RS1, an aluminum-tolerant *Basidiomycota* fungus, was determined and compared with the known mitochondrial genomes of 12 *Basidiomycota* species. The mitochondrial genome of *R. taiwanensis* RS1 is a circular DNA molecule of 40,392 bp and encodes the typical 15 mitochondrial proteins, 23 tRNAs, and small and large rRNAs as well as 10 intronic open reading frames. These genes are apparently transcribed in two directions and do not show syntenies in gene order with other investigated *Basidiomycota* species. The average G+C content (41%) of the mitochondrial genome of *R. taiwanensis* RS1 is the highest among the *Basidiomycota* species. Two introns were detected in the sequence of the *atp9* gene of *R. taiwanensis* RS1, but not in that of other *Basidiomycota* species. *Rhodotorula taiwanensis* is the first species of the genus *Rhodotorula* whose full mitochondrial genome has been sequenced; and the data presented here supply valuable information for understanding the evolution of fungal mitochondrial genomes and researching the mechanism of aluminum tolerance in microorganisms.

## Introduction

Due to several special characteristics of mitochondria, such as high copy number, apparent lack of recombination, rapid evolution, and maternal inheritance, mitochondrial DNA has been used as a potent tool to research the origin of organisms (Ingman et al. 2000) and to infer phylogeny of closely related species (Biswas et al. 2001). A vast number of mitochondrial sequences of various species have been deposited in public databases, although the number of fungal mitochondrial genome sequences is still limited. The “higher fungi” are the majority of fungi whose mitochondrial genomes have been sequenced. However, compared with 85 records of mitochondrial genomes from the ascomycetous yeasts, there are only 13 records of 12 species from another group of “higher fungi,” the so-called basidiomycetous yeasts, available at the GenBank database (http://www.ncbi.nlm.nih.gov/genomes/GenomesGroup.cgi?opt=organelle&taxid=451864; as of 21 November 2012), indicating a huge imbalance in the number of mitochondrial genome sequences between the two phyla, *Basidiomycota* and *Ascomycota*. Fell et al. (2000) pointed out that the basidiomycetous yeasts have considerable agricultural, industrial, environmental, and medical importance. The analysis of complete mitochondrial genomes of basidiomycetous yeasts provides not only a phylogenetically more balanced data set but also detailed information for understanding the origin and evolution of organisms.

Previously, we isolated an aluminum-tolerant strain from acidic soils and named it *Rhodotorula* sp. RS1 (=CGMCC 2.4753; Wang et al. 2013). *Rhodotorula* belongs to the basidiomycetous yeasts and has been found and isolated from air, soil, human skin, stool, and food (Biswas et al. 2001). However, the complete mitochondrial genome of the genus *Rhodotorula* has not been reported so far. In addition, recent research has revealed that mitochondria plays a crucial role in aluminum toxicity and tolerance in *Rhodotorula* yeast (Tani et al. 2008) as well as in plants (Yamamoto et al. 2002) and humans (Mailloux et al. 2007). Therefore, we determined the complete mitochondrial genome of *Rhodotorula* sp. RS1 and compared it with that of 12 other *Basidiomycota* mitochondrial genomes.

## Materials and Methods

### Culture condition and identification of the yeast

*Rhodotorula* sp. RS1 (=CGMCC 2.4753) was isolated from acidic oil–tea soils and is highly tolerant to aluminum toxicity (Wang et al. 2013). The strain was maintained and grown on glucose medium (Wang et al. 2013), and its physiological and morphological characteristics were investigated according to the methods described by Kurtzman et al. (2011).

### DNA preparation, sequencing, and assembly

The total genomic DNA of *Rhodotorula* sp. RS1 was extracted from the strain using QIAGEN Genomic-tips (QIAGEN, Hilden, Germany), Genomic DNA Buffer Set (QIAGEN), and Zymolyase-20T (Nacalai Tesque, Kyoto, Japan) as described by the manufacturers and sequenced by a whole-genome-shotgun strategy. One paired-end run was performed using a Roche (Basel, Switzerland) 454 Genome Sequencer (FLX Titanium). The GS FLX run resulted in the generation of about 1,089,220 reads with a total of 470,223,950 bases and an average length of 432 bp. Reads were assembled using the GS de novo Assembler 2.5 software program (454 Life Science, Branford, CT). Sequence coverage was 13.6×. We found that the length of the longest contig of the assembled sequences was similar to that of mitochondrial genomes of other fungus species. Subsequent analysis of genome assembly by use of Consed (Gordon et al. 1998) indicated that this contig was a circular DNA sequence. It was then identified as the mitochondrial genome of the fungus by comparison with other fungal mitochondrial genomes.

### Genome annotation

The mitochondrial genome sequence was first imported into a Rapid Annotation Platform for Yeast Data (RAPYD; Schneider et al. 2011). In RAPYD, the mitochondrial genome was first auto-annotated. The auto-annotation of the mitochondrial genome was manually checked and improved using two different applications, EXONERATE (Slater and Birney 2005) and MFannot (http://megasun.bch.umontreal.ca/cgi-bin/mfannot/mfannotInterface.pl). The start codons, stop codons, and exon–intron boundaries of protein-coding genes were modified through tblastn against the mitochondrial genome of *Rhodotorula* sp. RS1 using the related annotated protein sequences of three *Basidiomycota* species, *Phakopsora meibomiae* (NC_014352), *Rhodotorula glutinis* (AB248915), and *Ustilago maydis* (NC_008368). The exon–intron boundaries were further adjusted using GenomeView (Abeel et al. 2012), based on the rule that introns generally start with GT and end with AG. Genes encoding tRNAs were predicted with tRNAscan-SE (Schattner et al. 2005). Genes encoding rRNAs were validated through blastn against an rRNA database Rfam (Gardner et al. 2009) as suggested by Haas et al. (2011). Additional open reading frames (ORFs) were annotated by searching them against the protein family database Pfam (Punta et al. 2012).

The mitochondrial genomes and annotations of *Cryptococcus neoforman*s (NC_004336), *C. neoformans* (NC_018792), *Lentinula edodes* (NC_018365), *Moniliophthora perniciosa* (NC_005927), *M. roreri* (NC_015400), *Pleurotus ostreatus* (NC_009905), *Schizophyllum commune* (NC_003049), *Trametes cingulata* (NC_013933), *P. meibomiae* (NC_014352), *P. pachyrhizi* (NC_014344), *Tilletia indica* (NC_009880), *T. walkeri* (NC_010651), and *U. maydis* (NC_008368) are available at GenBank. Although there are two mitochondrial genome sequences of the same strain *C. neoformans* H99, NC_004336 and NC_018792, deposited in GenBank, the sequences are 100% identical except for an additional 45-bp direct repeat sequence in the NC_018792 at the position 1838 to 1882, which in addition caused little topological change in the phylogenetic trees determined in this present research. The mitochondrial genome sequence of *Rhodotorula* sp. RS1 has been submitted to EMBL-EBI database (HF558455).

The tandem repeats within the full mitochondrial genome sequence were identified using Tandem Repeats Finder (http://tandem.bu.edu/trf/trf.html) (Benson 1999). G+C contents of mitochondrial genomes were analyzed using BioEdit (Hall 1999) version 7.1.3.0. Codon usage was computed using codonw 1.4.4 through online website Browsers (http://mobyle.pasteur.fr/cgi-bin/portal.py?#forms::codonw).

### Phylogenetic analysis

Phylogenetic analysis was done mainly according to the methods described by Valach et al. (2011) with some modifications. The 15 or 14 common protein sequences among the fungal mitochondrial genomes were concatenated in the order of *cox1-cox2-cox3-cob-atp6-atp8-atp9-nad1-nad2-nad3-nad4-nad4L-nad5-nad6-*(*rps3*) using BioEdit (Hall 1999) version 7.1.3.0. *Smittium culisetae* (NC_006837) was used as the out-group to construct the phylogenic tree. The concatenated sequences were aligned using the MUSCLE (Edgar 2004) algorithm included in the MEGA5 package (Tamura et al. 2011) with default parameters. Phylogenetic analysis was performed by the maximum likelihood method based on the Whelan and Goldman (WAG; Whelan and Goldman 2001) and Jones–Taylor–Thornton (JTT; Jones et al. 1992) models provided in the MEGA5 package (Tamura et al. 2011). The bootstrap consensus tree inferred from 100 replicates was taken to represent the evolutionary history of the taxa analyzed (Felsenstein 1985). For the phylogenetic analysis of *Rhodotorula* species based on the D1/D2 domain, ITS region, and *cob* gene sequence, the unweighted pair group method with arithmetic mean (UPGMA) method included in the MEGA5 package (Tamura et al. 2011) was used, as Biswas et al. (2001) suggested that the UPGMA method is more suitable to construct the phylogenetic tree based on the *cob* gene sequence than other methods.

## Results

### Identification of *Rhodotorula* sp. RS1

In our previous report (Wang et al. 2013), phylogenetic analysis using 26S rRNA D1/D2 domain sequence showed that *Rhodotorula* sp. RS1 is closely related to *Rhodotorula taiwanensis* BCRC 23118^T^, which was identified as a novel *Rhodotorula* species by Huang et al. (2011). Further comparison of the nuclear rRNA sequence showed that *Rhodotorula* sp. RS1 and *R. taiwanensis* BCRC 23118^T^ differ by 1 and 3 nucleotides in the internal transcribed spacer (ITS) region and D1/D2 domain, respectively. Generally, species with differences of less than 1% in the D1/D2 domain or of 1–2% in the ITS region are recognized to be conspecific (Kurtzman and Robnett 1998; Hamamoto et al. 2002; Choudhary and Johri 2009; Huang et al. 2011). Phylogenetic analysis of the D1/D2 domain or ITS region of the type strains of *Rhodotorula* and *Rhodotorula* sp. RS1 revealed that *Rhodotorula* sp. RS1 formed single cluster with *R. taiwanensis* BCRC 23118^T^ but a separate line of descent in the phylogenetic cluster of other *Rhodotorula* species (Figs. S1 and S2). In addition, we also analyzed the physiological and morphological characteristics, such as nitrogen and carbon assimilation, of *Rhodotorula* sp. RS1, which showed similar results to those of *R. taiwanensis* BCRC 23118^T^ (Table S1). On the basis of these analyses, we concluded that *Rhodotorula* sp. RS1 belongs to the species *R. taiwanensis*. The type strain of *R. taiwanensis* showed growth on the glucose medium containing up to 200 mmol/L Al^3^^+^ as aluminum sulfate, indicating that both strains showed similar tolerance to aluminum.

### Gene contents

**Figure 1:**
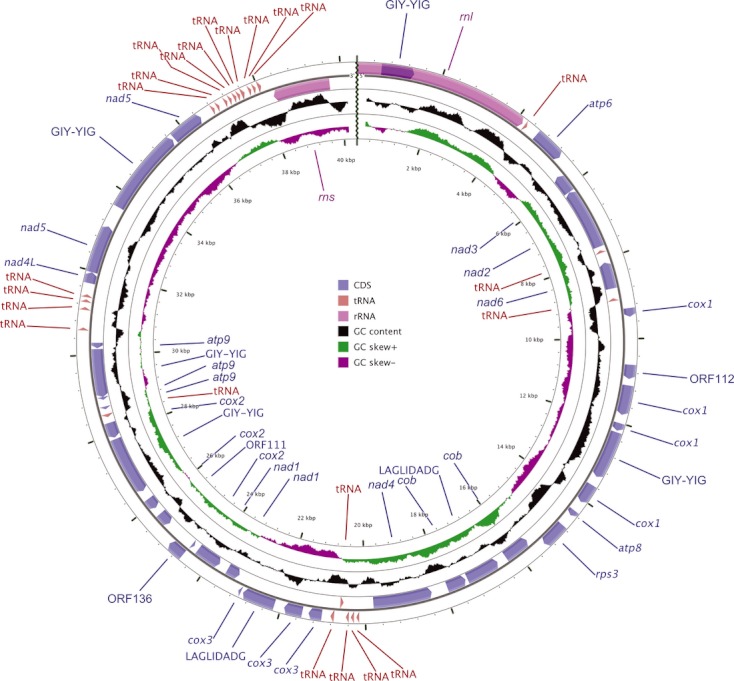
Physical map of the *Rhodotorula taiwanensis* RS1 mitochondrial genome. From the outside in, the various rings represent (i) *R*. *taiwanensis* RS1 genes identified in the clockwise strand; (ii) genes identified in the anti-clockwise strand; (iii) G+C content as the deviation from the average over the entire genome; and (iv) G+C skew as the deviation from the average over the entire genome. Unannotated open reading frames (ORFs) longer than 100-codons are depicted with the number of their corresponding amino acids in the labels. The image was generated by using CGVIEW (Grant and Stothard 2008).

The mitochondrial genome of *R. taiwanensis* RS1 was shown to be a circular DNA molecule of 40,392 bp with an average G+C content of 41% (Fig. 1 and Table 1). Fifty possible encoding regions were identified in the mitochondrial genome, including 15 typical mitochondrial protein-coding genes, two rRNAs, 23 tRNAs, and 10 intronic ORFs (Fig. 1 and Table S2). These genes are apparently transcribed from two different strands. Only one tandem repeat with the sequence “AGTACCTTGTGT” was identified at the position 7975 to 8021, which had a low copy number (3.8) with 91% as match percentage.

**Table 1:** General features of mitochondrial genomes of the *Basidiomycota*: *Rhodotorula taiwanensis* RS1 (Rt), *Phakopsora pachyrhizi* (Pp), *Phakopsora meibomiae* (Pm), *Tilletia indica* (Ti), *Tilletia walkeri* (Tw), *Ustilago maydis* (Um), *Cryptococcus neoforman*s (Cn), *Trametes cingulata* (Tc), *Schizophyllum commune* (Sc), *Pleurotus ostreatus* (Po), *Lentinula edodes* (Le), *Moniliophthora perniciosa* (Mp), and *M. roreri* (Mr)

	Features				
	
Species	Genome size (bp)	G+C (%)	Number of tRNA	Number of CDS	Protein-coding regions (%)
Rt	40,392	41	23 (4^1^)	25 (12^1^)	56
Pp	31,825	35	24 (0)	15 (0)	41
Pm	32,520	35	24 (0)	15 (0)	40
Ti	65,147	29	24 (8)	15^2^ (6)	20
Tw	59,352	29	24 (9)	15^2^ (6)	22
Um	56,814	31	23 (9)	26 (13)	38
Cn	24,874	35	21 (1)	16 (0)	57
Tc	91,500	24	25 (1)	20 (2)	19
Sc	49,704	22	27 (11)	20 (4)	47
Po	73,242	26	24 (1)	26 (1)	39
Le	121,394	31	28 (2)	27 (1)	23
Mp	109,103	32	26 (0)	89 (23)	52
Mr	93,722	28	26 (1)	56 (18)	45

1 The number within the bracket indicates the number of genes encoded on a reverse strand.

2 The *rps3* gene was detected in this study.

Among the 15 typical protein-coding genes in the mitochondrial genome of *R*. *taiwanensis* RS1 (Fig. 1 and Table S2), 14 are involved in oxidative and energy metabolism: three ATP synthase subunits, 6, 8, and 9; three cytochrome oxidase subunits, 1–3; seven NADH dehydrogenase subunits, 1–6 and 4L; and one cytochrome b. The remaining one was annotated as ribosomal small subunit protein 3 gene (*rps3*), which participates in ribosome assembly (Bullerwell et al. 2000).

Thirteen introns were identified in the mitochondrial genome of *R*. *taiwanensis* RS1: three introns in *cox1*, two introns each in *cox2*, *cox3*, and *atp9*, one intron each in *nad1*, *nad5*, *cob*, and *rnl* (Fig. 1 and Table 2). Ten intronic ORFs were located inside the seven genes that coded for *rnl* (1), *cox1* (2), *cob* (1), *cox3* (1), *cox2* (3), *atp9* (1), and *nad5* (1; Fig. 1 and Table S2). Five intronic ORFs located inside the genes *rnl*, *cox1*, *cox2*, *atp9*, and *nad5* showed amino acid sequence similarity to the GIY-YIG motif of homing endonucleases; whereas two intronic ORFs located inside *cob* and *cox3* exhibited similarity to the LAGLIDADG motif. The remaining three intronic ORFs located inside *cox1* and *cox2* did not reveal any significant amino acid sequence similarity to known genes.

**Table 2:** Number of introns found in the mitochondrial genomes of the *Basidiomycota*

	Number of introns												
	
Genes	Rt^1^	Pp	Pm	Ti	Tw	Um	Cn	Tc	Sc	Po	Le	Mp	Mr
*cox1*	3	3	3	5	4	8	0	15	0	9	7	6	5
*cox2*	2	0	0	0	0	0	0	2	0	1	0	2	0
*cox3*	2	0	0	0	0	0	0	0	0	0	1	0	0
*atp6*	0	0	0	0	0	0	0	0	0	0	0	0	0
*atp8*	0	0	0	0	0	0	0	0	0	0	0	0	0
*atp9*	2	0	0	0	0	0	0	0	0	0	0	0	0
*nad1*	1	0	0	1	1	0	1	0	0	0	3	1	3
*nad2*	0	0	0	0	0	0	0	0	0	1	0	0	0
*nad3*	0	0	0	0	0	0	0	0	0	0	0	0	0
*nad4*	0	0	0	1	1	0	0	0	0	1	0	1	0
*nad4L*	0	0	0	0	0	0	0	0	0	0	0	0	0
*nad5*	1	0	0	2	2	1	0	2	0	0	1	1	2
*nad6*	0	0	0	0	0	0	0	0	0	0	0	0	0
*rps3*	0	0	0	0	0	0	0	0	0	0	0	0	0
*cob*	1	1	1	1	0	1	1	0	0	0	3	2	2
*rnl*	1	1	1	0	1	2	0	6	0	0	3	0	0
*rns*	0	0	0	0	1	0	0	1	0	0	1	0	0

1 Abbreviations are the same as in Table 1.

The 23 identified tRNA genes in the mitochondrial genome of *R*. *taiwanensis* RS1 represented all 20 amino acids and included two copies encoding tRNA^Met^, tRNA^Ser^, and tRNA^Leu^ (Fig. 1 and Table S2). Single-copy genes encoded the remaining 17 tRNAs. The tRNA genes were located on the two different strands. Nineteen of the tRNAs were grouped into three clusters of 10, 4, and 5 tRNA genes each; whereas the other four tRNA genes occurred singly. The large and small subunit rRNA genes (*rnl* and *rns*) were located next to each other but on different strands.

### Codon usage

The preferential codon usage was calculated from the exon sequences of the 15 typical mitochondrial protein-coding genes and 10 intronic ORFs (Table S3). Three codons, ATA, CCC, and AGG, were not used for the 15 typical protein-coding genes; whereas all possible codons were used at least two times for the 10 intronic ORFs. For the 15 typical protein-encoding genes, the five most frequent amino acids were leucine, valine, alanine, serine, and isoleucine; and the five most abundant codons were CTA, TTC, GTA, GCT, and ATT. For the 10 intronic ORF genes, the five most frequent amino acids were leucine, serine, threonine, valine, and alanine; and the five most abundant codons were ACT, GTA, ATT, TTA, and ACA. With the exception of isoleucine, alanine, glutamic acid, and cysteine, the codons corresponding to mitochondrial tRNAs were used more frequently than other codons in the 15 typical protein-coding genes. However, in the 10 intronic ORF genes, most of amino acids did not show a preference for the mitochondrial tRNA codons over other codons.

In the mitochondrial genome of *R*. *taiwanensis* RS1, all protein-coding sequences started with the ATG codon with the exception of *cox1* and two intronic genes encoding GIY-YIG and LAGLIDADG endonucleases starting with the codons TTG, ATT, and ATC, respectively. All protein-coding sequences used TAA or TAG as the stop codon. The alternate codon TGA for tryptophan, which differs from the tryptophan codon of the standard genetic code, did not represent a stop codon in all protein-coding genes. TGA was present eight times in the 10 intronic ORF genes to code for tryptophan but only once in the 15 typical protein-coding genes that used TGG to code for tryptophan 73 times, indicating a strong Try_TGG_ bias in the mitochondrial genome of *R*. *taiwanensis* RS1.

### Comparative genomics

*Rhodotorula taiwanensis* RS1 had a relatively small mitochondrial genome compared with other *Basidiomycota* species, whereas its mitochondrial genome was characterized by the highest G+C content among the *Basidiomycota* (Table 1). The protein-coding and noncoding regions of the mitochondrial genome of *R*. *taiwanensis* RS1 had similar G+C contents of 41% and 40%, respectively.

At GenBank, all the *Basidiomycota* contained 14 conserved protein-coding genes involved in the respiratory mechanism. The gene *rps3*, coding for ribosomal protein, was not annotated in *T. indica* (NC_009880) and *T. walkeri* (NC_010651), whereas the remaining 10 *Basidiomycota* species contained the *rps3* gene. However, we found two homologous regions of *rps3* in the two *Tilletia* species by a blast search against their mitochondrial genome sequences using the *rps3* sequences of other *Basidiomycota* species. Simultaneously, two ORFs were annotated in the same homologous regions of *T. indica* (position: 33,848..35,035, complement) and *T. walkeri* (position: 32,601..33,788, complement) using MFannot software. Furthermore, the two ORFs showed amino acid sequence similarity to the ribosomal protein S3 motif by searching for them against the protein-family database Pfam. These results suggested that the mitochondrial genomes of *T. indica* and *T. walkeri* also contained *rps3* genes. Thus, all the *Basidiomycota* species contain a standard set of 15 common protein-coding genes in their mitochondrial genomes.

The large and small rRNAs existed in the mitochondrial genomes of all the *Basidiomycota* species. The number of tRNAs among the mitochondrial genomes of the *Basidiomycota* species ranged from 21 to 28, and 23 tRNAs in *R. taiwanensis* RS1 were within this range (Table 1). The protein number (25) of the mitochondrial genome of *R. taiwanensis* RS1 was moderate among the *Basidiomycota* species, which varied from 15 in *Tilletia* species to 89 in *M. perniciosa* (Table 1). The protein-coding regions of *R. taiwanensis* RS1 accounted for 56% of the total mitochondrial genome, which was the highest portion among the *Basidiomycota* species except *C. neoforman*s (Table 1).

The position and number of introns were not conserved among these *Basidiomycota* species (Table 2). The gene *cox1* was characterized by more introns relative to other genes with the exception of *C. neoformans* and *S. commune*. Introns were absent in the genes *atp6*, *atp8*, *nad3*, *nad4L*, *nad6*, and *rps3* of all *Basidiomycota* species presented here. It is noticeable that there were two introns in the *atp9* of *R*. *taiwanensis* RS1 but not in other *Basidiomycota* species. Introns were identified in *cox3* of only *R*. *taiwanensis* RS1 and *L. edodes* but not in other *Basidiomycota* species.

**Figure 2:**
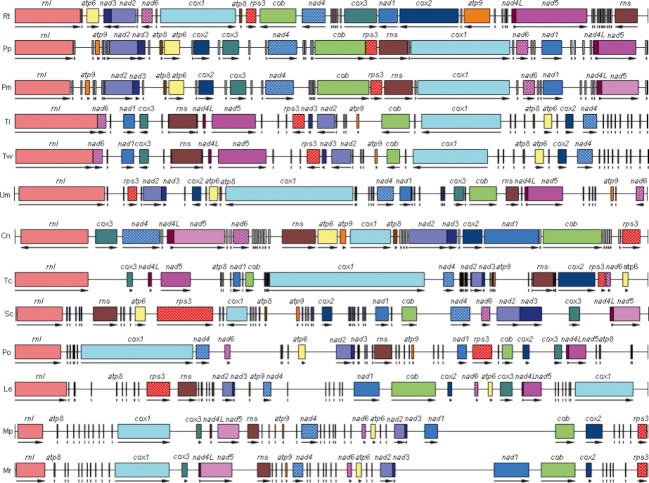
Comparison of the gene content and order of the known mitochondrial genomes from *Basidiomycota*. This map was drawn by using the PlasDraw software contained in GENETYX version 10. Abbreviations are the same as in Table 1. Gene sizes and total mitochondrial genome length were drawn to match those of the genome of *Rhodotorula taiwanensis* RS1. Black vertical lines represent tRNAs that were not labeled.

All the *Basidiomycota* mitochondrial genomes investigated here presented a circular topology. *Rhodotorula taiwanensis* RS1 did not show synteny with other *Basidiomycota* species in the order of the protein-coding and RNA genes (Fig. 2). The species belonging to different genera did not exhibit overall synteny in gene order among the *Basidiomycota* species. However, consistent gene orders between *P. meibomiae* and *P. pachyrhizi*, *T. indica* and *T. walkeri*, and *M. perniciosa* and *M. roreri* were observed. These findings are in accordance with their classifications under the same taxonomical genus: *Phakopsora*, *Tilletia*, and *Moniliophthora*, respectively.

While gene order did not exhibit synteny among *Basidiomycota*, several genes were clustered together on the genome. In all 13 species examined, *nad4L* and *nad5* were clustered together in the order of *nad4L*-*nad5*. In *R*. *taiwanensis* RS1, *T. indica* and *T. walkeri*, *nad2* and *nad3* were together in the order of *nad3*-*nad2*; but they occurred in the order of *nad2*-*nad3* in the remaining 10 species.

### Phylogenetic analysis

**Figure 3:**
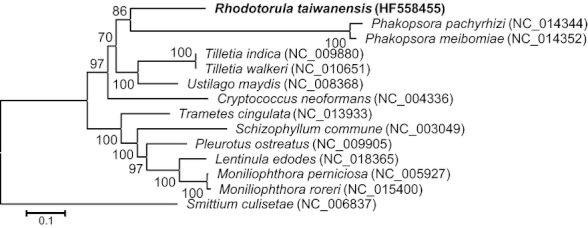
Molecular phylogenetic analysis of *Rhodotorula taiwanensis* RS1 and other *Basidiomycota* species by use of the amino acid sequences of 15 common protein-coding genes and the maximum likelihood method based on the Whelan and Goldman (WAG) model. Numbers at branch nodes are percentages based on 100 bootstrap resampling; only values over 50% are given. Bar, 0.1 substitutions per nucleotide position.

When the phylogenetic relationship of *R*. *taiwanensis* RS1 to other *Basidiomycota* species was examined in terms of the amino acid sequence of the 15 common protein-coding genes based on the WAG model, *R*. *taiwanensis* RS1 and the two *Phakopsora* species were clustered in the same clade (Fig. 3). This finding was further confirmed using the JTT model (Fig. S3). As the sequence of the *rps3* gene was not very conserved among the *Basidiomycota* species, we further constructed a phylogenetic tree using the amino acid sequences of all common protein-coding genes except *rps3*. The obtained results were very similar to those using the 15 common protein-coding genes (Fig. S4).

## Discussion

Tani et al. (2008) determined the sequence of several genes of the mitochondrial genome of *R. glutinis* (AB248915). In addition, the DOE Joint Genome Institute announced the mitochondrial genome sequence of *R. graminis* on its website (http://genome.jgi.doe.gov/Rhoba1_1/Rhoba1_1.home.html), but it was not annotated and may also be an incomplete sequence according to our analysis. Thus, to the best of our knowledge, the present study represents the first report of complete annotated mitochondrial genome sequence of the genus *Rhodotorula*. The number of mitochondrial genome sequences within the phylum *Basidiomycota* available at GenBank will be 14 with the addition of the mitochondrial genome sequence of *R*. *taiwanensis* RS1.

The yeast genus *Rhodotorula* is greatly marked by its prominent polyphyly, as it is distributed over four classes, that is, *Microbotryomycetes*, *Cystobasidiomycetes*, *Ustilaginomycetes*, and *Exobasidiomycetes*, in two subphyla, *Pucciniomycotina* and *Ustilaginomycotina* (Sampaio 2011). The phylogenetic analysis of mitochondrial genome data here demonstrates that *R*. *taiwanensis* RS1 was clustered together with the two *Phakopsora* species belonging to the subphylum *Pucciniomycotina* (Stone et al. 2010). Biswas et al. (2001) demonstrated that the sequence of the mitochondrial cytochrome *b* gene, *cob*, is effective to identify species and study phylogenetic relationships among basidiomycetous yeasts. Therefore, we constructed the phylogenetic trees based on the DNA sequence or amino acid sequence of the mitochondrial gene *cob* of *R*. *taiwanensis* RS1 and other *Rhodotorula* species (Figs. S5 and S6, respectively). Topologies of these trees were similar not only to each other but also to that of the phylogenetic trees of the sequences of nuclear D1/D2 and ITS (Figs. S1 and S2, respectively). *Rhodotorula taiwanensis* RS1 was clustered together with *R. dairenensis* and *R. mucilaginosa* in the same clade in these four trees (Figs. S1, S2, S5, and S6). There were some inconsistencies among the phylogenetic trees of D1/D2, ITS, and *cob* of this study, which have been recognized in previous reports (Biswas et al. 2001; Scorzetti et al. 2002). Therefore, the physiological and morphologic characteristics are also important for identifying the taxonomic assignment of novel species.

Despite the tremendous variation in size of the mitochondrial genome among the basidiomycetous yeasts (Table 1), we found 15 common protein-coding genes, small and large rRNAs, and more than 21 tRNAs in all *Basidiomycota* mitochondria genomes completely sequenced so far. It is well known that there is no apparent correlation between mitochondrial genome size and gene content among the basidiomycetous yeasts (Formighieri et al. 2008; Haridas and Gantt 2010; Stone et al. 2010), as some other factors such as intergenic spacers, introns, undetermined ORFs, and integrated plasmids result in the variation in mitochondrial genome size.

In agreement with previous reports (Formighieri et al. 2008; Stone et al. 2010; Costa et al. 2012), an overall synteny in the gene order of mitochondrial genome was not observed among the *Basidiomycota* species of different genera; whereas those species within the same genus presented a high degree of synteny. These results suggest that gene shuffling events may scarcely occur in the same *Basidiomycota* genus and that gene shuffling events may be involved in the emergence of new genera. Even in the absence of overall synteny, the present study further supports the two linkages between *nad* genes (*nad2*-*nad3* and *nad4L*-*nad5*) in the *Basidiomycota* during their evolution (Formighieri et al. 2008; Wang et al. 2008; Stone et al. 2010).

Although mitochondrial genomes of most fungi are A+T biased that of *R*. *taiwanensis* RS1 showed a relatively less A+T biased than other members of the *Basidiomycota*. The G+C contents of partial mitochondrial genome sequences of *R. glutinis* (AB248915) and *R. graminis* (http://genome.jgi.doe.gov/Rhoba1_1/Rhoba1_1.home.html) were also a relatively less A+T biased (38%), suggesting the mitochondrial genome of *Rhodotorula* may have evolved with a weaker mutational bias toward A and T in comparison with other known *Basidiomycota* members.

Interestingly, two introns were identified in the *atp9* gene of *R*. *taiwanensis* RS1 but not in that of other *Basidiomycota*. There is only one intron in the *atp9* gene of *R*. *glutinis* IFO1125 (AB248915). The insertion of an additional intron into the *atp9* gene of the mitochondrial genome of *R*. *taiwanensis* RS1 may represent a specific feature during its evolution compared with that of other *Basidiomycota* species.

Many aluminum-tolerant ascomycetous and basidiomycetous yeasts have been isolated from soil (Kanazawa and Kunito 1996; Kawai et al. 2000; Kunito et al. 2012). The observed increases in mitochondrial number and copy number of mitochondrial DNA were suggested to be involved in the aluminum tolerance of *R. glutinis* as a compensatory response to reduced respiratory activity caused by a deficiency in complex IV function (Tani et al. 2008). The inhibitory influence of aluminum on ATP production and on mitochondrial functions has been reported as causes for aluminum-triggered neurological disorders (Lemire and Appanna 2011). Our previous report suggested that the thickening of the cell wall may be involved in the high aluminum tolerance of *R. taiwanensis* RS1 (Wang et al. 2013). The mitochondrial genomics survey of aluminum-tolerant *R*. *taiwanensis* RS1 presented here can facilitate the identification of key genes involved in aluminum tolerance, which would then provide knowledge for the bioremediation and improvement of acidic soils, especially as aluminum toxicity is the primary factor limiting agricultural production in acidic soils (Kochian et al. 2004).

In conclusion, the present study reports the first complete mitochondrial genome sequence of the genus *Rhodotorula*. The mitochondrial genome of *R*. *taiwanensis* RS1 was shown to be a circular DNA molecule containing 15 common mitochondrial genes, in which finding is in accordance with reported mitochondrial genomes of other basidiomycetous yeasts; although it did not show any syntenies in gene order with other basidiomycetous yeasts. A higher G+C content and two introns included in the *atp9* gene were two unique characteristics of *R*. *taiwanensis* RS1, making it different from other basidiomycetous yeasts. These results supply basic information for researching the evolution of fungal mitochondrial genomes and the mechanism of aluminum tolerance in basidiomycetous yeasts.

## Supporting Information

**Figure S1.** Molecular phylogenetic analysis of *Rhodotorula taiwanensis* RS1 and other *Rhodotorula* species by using the D1/D2 domain sequence. The analysis was performed as described by Biswas et al. (2001) by using the unweighted pair group method with arithmetic mean (UPGMA) method included in the MEGA5 package (Tamura et al. 2011) with default parameters. T, type strain. *Candida glabrata* was used as the out-group. (A) Class *Microbotryomycetes*; (B) Class *Cystobasidiomycetes*; and (C) Class *Exobasidiomycetes*, which were classified according to Sampaio (2011). Numbers at branch nodes are percentages based on 100 bootstrap resampling; only values over 50% are given. Bar, 0.02 substitutions per nucleotide position.

**Figure S2.** Molecular phylogenetic analysis of *Rhodotorula taiwanensis* RS1 and other *Rhodotorula* species by using internal transcribed spacer (ITS) region sequence. The analysis was performed as described in Figure S1. T, type strain. *Candida glabrata* was used as the out-group. A, B, C, and numbers at branch nodes are the same as in Figure S1. Bar, 0.02 substitutions per nucleotide position.

**Figure S3.** Molecular phylogenetic analysis of *Rhodotorula taiwanensis* RS1 and other *Basidiomycota* species conducted by using 15 common protein-coding amino acid sequences and the maximum likelihood method based on the Jones–Taylor–Thornton (JTT) model. Numbers at branch nodes are the same as in Figure S1. Bar, 0.1 substitutions per nucleotide position.

**Figure S4.** Molecular phylogenetic analysis of *Rhodotorula taiwanensis* RS1 and other *Basidiomycota* species based on 14 common protein-coding amino acid sequences and the maximum likelihood method applied to the WAG (A) and JTT (B) models. Numbers at branch nodes are the same as in Figure S1. Bars, 0.1 substitutions per nucleotide position.

**Figure S5.** Molecular phylogenetic analysis of *Rhodotorula taiwanensis* RS1 and other *Rhodotorula* species based on nucleotide sequences of coding regions of the *cob* gene. This analysis was conducted as described in Figure S1. T, type strain; and NT, neotype strain. *Candida glabrata* was used as the out-group. A, B, C, and numbers at branch nodes are the same as in Figure S1. Bar, 0.02 substitutions per nucleotide position.

**Figure S6.** Molecular phylogenetic analysis of *Rhodotorula taiwanensis* RS1 and other *Rhodotorula* species based on amino acid sequences of coding regions of the *cob* gene. This analysis was conducted as described in Figure S1. T, type strain; and NT, neotype strain. *Candida glabrata* was used as the out-group. A, B, C, and numbers at branch nodes are the same as in Figure S1. Bar, 0.02 substitutions per nucleotide position.
