# Supplementary material for: Complete mitochondrial genome of the aluminum-tolerant fungus Rhodotorula taiwanensis RS1 and comparative analysis of Basidiomycota mitochondrial genomes
Source: Microbiologyopen. 2013 Feb 21;2(2):308–17. doi: 10.1002/mbo3.74 (PMC3633354; doi:10.1002/mbo3.74)
Supplement: Supplementary file 1 [file mbo30002-0308-SD1.doc]

**Figure legends**

**Fig. S1.** Molecular phylogenetic analysis of *R*. *taiwanensis* RS1 and other *Rhodotorula* species by using the D1/D2 domain sequence. The analysis was performed as described by Biswas *et al*. (2001) by using the UPGMA method included in the MEGA5 package (Tamura *et al*., 2011) with default parameters. T, type strain. *Candida glabrata* was used as the out-group. A, Class *Microbotryomycetes*; B, Class *Cystobasidiomycetes*; and C, Class *Exobasidiomycetes*, which were classified according to Sampaio (2011). Numbers at branch nodes are percentages based on 100 bootstrap resampling; only values over 50% are given. Bar, 0.02 substitutions per nucleotide position.

**Fig. S2.** Molecular phylogenetic analysis of *R*. *taiwanensis* RS1 and other *Rhodotorula* species by using ITS region sequence. The analysis was performed as described in Fig. S1. T, type strain. *Candida glabrata* was used as the out-group. A, B, and C are the same as in Fig. S1. Numbers at branch nodes are the same as in Fig. S1. Bar, 0.02 substitutions per nucleotide position.

**Fig. S3.** Molecular phylogenetic analysis of *R*. *taiwanensis* RS1 and other *Basidiomycota* species conducted by using 15 common protein-coding amino acid sequences and the Maximum Likelihood method based on the JTT model. Numbers at branch nodes are the same as in Fig. S1. Bar, 0.1 substitutions per nucleotide position.

**Fig. S4.** Molecular phylogenetic analysis of *R*. *taiwanensis* RS1 and other *Basidiomycota* species based on 14 common protein-coding amino acid sequences and the Maximum Likelihood method applied to the WAG (a) and JTT (b) models. Numbers at branch nodes are the same as in Fig. S1. Bars, 0.1 substitutions per nucleotide position.

**Fig. S5.** Molecular phylogenetic analysis of *R*. *taiwanensis* RS1 and other *Rhodotorula* species based on nucleotide sequences of coding regions of the *cob* gene. This analysis was conducted as described in Fig. S1. T, type strain; and NT, neotype strain. *Candida glabrata* was used as the out-group. A, B, C, and numbers at branch nodes are the same as in Fig. S1. Bar, 0.02 substitutions per nucleotide position.

**Fig. S6.** Molecular phylogenetic analysis of *R*. *taiwanensis* RS1 and other *Rhodotorula* species based on amino acid sequences of coding regions of the *cob* gene. This analysis was conducted as described in Fig. S1. T, type strain; and NT, neotype strain. *Candida glabrata* was used as the out-group. A, B, C, and numbers at branch nodes are the same as in Fig. S1. Bar, 0.02 substitutions per nucleotide position.
