# Supplementary material for: Complete mitochondrial genome of the aluminum-tolerant fungus Rhodotorula taiwanensis RS1 and comparative analysis of Basidiomycota mitochondrial genomes
Source: Microbiologyopen. 2013 Feb 21;2(2):308–17. doi: 10.1002/mbo3.74 (PMC3633354; doi:10.1002/mbo3.74)
Supplement: Supplementary file 2 [file mbo30002-0308-SD2.doc]

**Table S1.** Comparison of physiological characteristics of *Rhodotorula* sp. RS1 and *R. taiwanensis* BCRC 23118^T^

| Characteristics | *Rhodotorula* sp. RS1 | *R. taiwanensis* BCRC 23118^T^ |
| --- | --- | --- |
| Assimilation of |  |  |
| Sorbose | + | + |
| Methyl α-D-glucoside | - | - |
| Ethanol | + | - |
| Nitrate | + | + |
| Nitrite | - | - |
| Ethylamine | + | + |
| _L_-Lysine | - | - |
| Cadaverine | + | + |
| Growth with |  |  |
| 35^0^C | W | + |
| 50% D-Glucose | + | + |

+, positive; -, negative; W, weak growth. The data of *R. taiwanensis* BCRC 23118^T^ were taken from Huang *et al*. (2011).

**Table S2.** Gene content of *R. taiwanensis* RS1 mitochondrial genome

| Gene | Position | Length (bp) | Orientation | Qualifiers |
| --- | --- | --- | --- | --- |
| *rnl* | 1-4159 | 4159 | forward | Large subunit ribosomal RNA |
| Intronic ORF | 576-1352 | 777 | forward | GIY-YIG endonuclease |
| *tRNA-P* | 4220-4291 | 72 | forward | Anticodon TGG |
| *atp6* | 4577-5338 | 762 | forward | ATP synthase subunit 6 |
| *nad3* | 5652-6131 | 480 | reverse | NADH dehydrogenase subunit 3 |
| *nad2* | 6137-7672 | 1536 | reverse | NADH dehydrogenase subunit 2 |
| *tRNA-G* | 7762-7832 | 71 | reverse | Anticodon TCC |
| *nad6* | 8083-8751 | 669 | reverse | NADH dehydrogenase subunit 6 |
| *tRNA-N* | 8989-9060 | 72 | reverse | Anticodon GTT |
| *cox1* | 9280-14068 | 4789 | forward | Cytochrome c oxidase subunit 1 |
| Intronic ORF | 10610-10948 | 339 | forward | Hypothetical protein |
| Intronic ORF | 12228-13373 | 1146 | forward | GIY-YIG endonuclease |
| *atp8* | 14318-14464 | 147 | forward | ATP synthase subunit 8 |
| *rps3* | 14757-15371 | 615 | forward | Ribosomal protein S3 |
| *cob* | 15627-17923 | 2297 | reverse | Apocytochrome b |
| Intronic ORF | 16529-17383 | 855 | reverse | LAGLIDADG endonuclease |
| *nad4* | 18322-19794 | 1473 | reverse | NADH dehydrogenase subunit 4 |
| *tRNA-R* | 20146-20216 | 71 | forward | Anticodon TCG |
| *tRNA-C* | 20269-20344 | 76 | forward | Anticodon GCA |
| *tRNA-W* | 20390-20462 | 73 | forward | Anticodon CCA |
| *tRNA-L* | 20527-20609 | 83 | reverse | Anticodon TAA |
| *tRNA-S* | 20738-20823 | 86 | forward | Anticodon TGA |
| *cox3* | 21025-23075 | 2051 | forward | cytochrome c oxidase subunit 3 |
| Intronic ORF | 22165-22953 | 789 | forward | LAGLIDADG endonuclease |
| *nad1* | 23213-24412 | 1200 | reverse | NADH dehydrogenase subunit 1 |
| *cox2* | 24549-28291 | 3743 | reverse | Cytochrome c oxidase subunit 2 |
| Intronic ORF | 24623-25033 | 411 | forward | Hypothetical protein |
| Intronic ORF | 25368-25703 | 336 | reverse | Hypothetical protein |
| Intronic ORF | 26405-27862 | 1458 | reverse | GIY-YIG endonuclease |
| *tRNA-L* | 28434-28516 | 83 | reverse | Anticodon TAG |
| *atp9* | 28657-30297 | 1641 | reverse | ATP synthase subunit 9 |
| Intronic ORF | 28946-30142 | 1197 | reverse | GIY-YIG endonuclease |
| *tRNA-H* | 30586-30656 | 71 | forward | Anticodon GTG |
| *tRNA-M* | 31067-31138 | 72 | forward | Anticodon CAT |
| *tRNA-I* | 31250-31320 | 71 | forward | Anticodon GAT |
| *tRNA-D* | 31368-31440 | 73 | forward | Anticodon GTC |
| *nad4L* | 31703-31966 | 264 | forward | NADH dehydrogenase subunit 4L |
| *nad5* | 31967-36496 | 4530 | forward | NADH dehydrogenase subunit 5 |
| Intronic ORF | 33614-35653 | 2040 | forward | GIY-YIG endonuclease |
| *tRNA-V* | 36808-36879 | 72 | forward | Anticodon TAC |
| *tRNA-M* | 36940-37010 | 71 | forward | Anticodon CAT |
| *tRNA-E* | 37152-37222 | 71 | forward | Anticodon TTC |
| *tRNA-T* | 37269-37339 | 71 | forward | Anticodon TGT |
| *tRNA-Q* | 37390-37461 | 72 | forward | Anticodon TTG |
| *tRNA-K* | 37488-37559 | 72 | forward | Anticodon TTT |
| *tRNA-S* | 37591-37676 | 86 | forward | Anticodon GCT |
| *tRNA-A* | 37776-37846 | 71 | forward | Anticodon TGC |
| *tRNA-F* | 37889-37960 | 72 | forward | Anticodon GAA |
| *tRNA-Y* | 38011-38094 | 84 | forward | Anticodon GTA |
| *rns* | 38295-39714 | 1420 | reverse | Small subunit ribosomal RNA |

**Table S3.** Codon usage for the 15 common mitochondrial protein-coding genes and ten intronic ORFs from the mitochondrial genome of *R.* *taiwanensis* RS1.

| Codon | Amino acid | N1^a^ | N2^b^ |  | Codon | Amino acid | N1 | N2 |  | Codon | Amino acid | N1 | N2 |  | Codon | Amino acid | N1 | N2 |
| --- | --- | --- | --- | --- | --- | --- | --- | --- | --- | --- | --- | --- | --- | --- | --- | --- | --- | --- |
| TTT | Phe | 45 | 56 |  | TCT | Ser | 58 | 79 |  | TAT | Tyr | 19 | 67 |  | TGT | Cys | 25 | 20 |
| TTC^*^ | Phe | 286 | 77 |  | TCC | Ser | 1 | 11 |  | TAC^*^ | Tyr | 181 | 86 |  | TGC^*^ | Cys | 2 | 15 |
| TTA^*^ | Leu | 101 | 100 |  | TCA^*^ | Ser | 178 | 72 |  | TAA | - | 10 | 4 |  | TGA | Trp | 1 | 8 |
| TTG | Leu | 1 | 44 |  | TCG | Ser | 2 | 12 |  | TAG | - | 5 | 6 |  | TGG^*^ | Trp | 73 | 31 |
| CTT | Leu | 146 | 50 |  | CCT | Pro | 85 | 92 |  | CAT | His | 12 | 57 |  | CGT | Arg | 21 | 61 |
| CTC | Leu | 3 | 24 |  | CCC | Pro | 0 | 6 |  | CAC^*^ | His | 94 | 47 |  | CGC | Arg | 1 | 10 |
| CTA^*^ | Leu | 367 | 92 |  | CCA^*^ | Pro | 85 | 47 |  | CAA^*^ | Gln | 98 | 71 |  | CGA^*^ | Arg | 84 | 44 |
| CTG | Leu | 38 | 37 |  | CCG | Pro | 6 | 5 |  | CAG | Gln | 13 | 35 |  | CGG | Arg | 3 | 13 |
| ATT | Ile | 194 | 105 |  | ACT | Thr | 116 | 116 |  | AAT | Asn | 9 | 88 |  | AGT | Ser | 60 | 58 |
| ATC^*^ | Ile | 151 | 60 |  | ACC | Thr | 1 | 16 |  | AAC^*^ | Asn | 108 | 79 |  | AGC^*^ | Ser | 72 | 27 |
| ATA | Ile | 0 | 5 |  | ACA^*^ | Thr | 161 | 93 |  | AAA^*^ | Lys | 55 | 76 |  | AGA | Arg | 1 | 2 |
| ATG^*^ | Met | 148 | 75 |  | ACG | Thr | 2 | 19 |  | AAG | Lys | 22 | 78 |  | AGG | Arg | 0 | 2 |
| GTT | Val | 105 | 51 |  | GCT | Ala | 207 | 82 |  | GAT | Asp | 47 | 77 |  | GGT | Gly | 138 | 86 |
| GTC | Val | 5 | 25 |  | GCC | Ala | 48 | 28 |  | GAC^*^ | Asp | 63 | 61 |  | GGC | Gly | 2 | 16 |
| GTA^*^ | Val | 252 | 113 |  | GCA^*^ | Ala | 113 | 68 |  | GAA^*^ | Glu | 33 | 65 |  | GGA^*^ | Gly | 175 | 41 |
| GTG | Val | 25 | 39 |  | GCG | Ala | 12 | 6 |  | GAG | Glu | 83 | 57 |  | GGG | Gly | 9 | 23 |

^a^number of codons of 15 common protein-coding genes; ^b^number of codons of 10 intronic ORFs;^*^ codons corresponding to the mitochondrial tRNA genes.
