# Supplementary material for: Complete mitochondrial genome of the aluminum-tolerant fungus Rhodotorula taiwanensis RS1 and comparative analysis of Basidiomycota mitochondrial genomes
Source: Microbiologyopen. 2013 Feb 21;2(2):308–17. doi: 10.1002/mbo3.74 (PMC3633354; doi:10.1002/mbo3.74)
Supplement: Supplementary file 3 [file mbo30002-0308-SD3.ppt]

## Slide 1
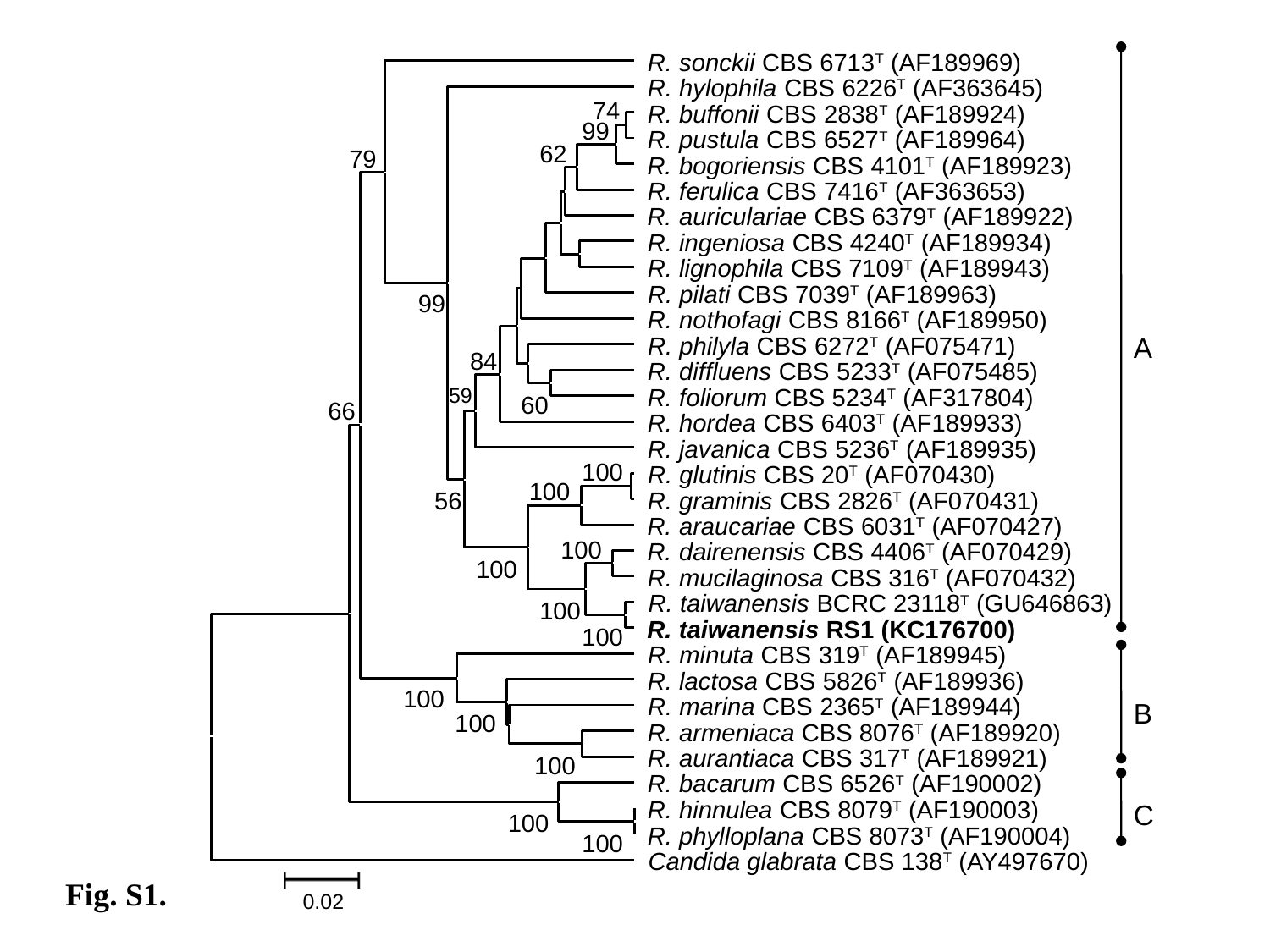

R. sonckii CBS 6713T (AF189969)
 R. hylophila CBS 6226T (AF363645)
74
 R. buffonii CBS 2838T (AF189924)
99
 R. pustula CBS 6527T (AF189964)
62
79
 R. bogoriensis CBS 4101T (AF189923)
 R. ferulica CBS 7416T (AF363653)
 R. auriculariae CBS 6379T (AF189922)
 R. ingeniosa CBS 4240T (AF189934)
 R. lignophila CBS 7109T (AF189943)
 R. pilati CBS 7039T (AF189963)
99
 R. nothofagi CBS 8166T (AF189950)
A
 R. philyla CBS 6272T (AF075471)
84
 R. diffluens CBS 5233T (AF075485)
59
 R. foliorum CBS 5234T (AF317804)
60
66
 R. hordea CBS 6403T (AF189933)
 R. javanica CBS 5236T (AF189935)
100
 R. glutinis CBS 20T (AF070430)
100
56
 R. graminis CBS 2826T (AF070431)
 R. araucariae CBS 6031T (AF070427)
100
 R. dairenensis CBS 4406T (AF070429)
100
 R. mucilaginosa CBS 316T (AF070432)
 R. taiwanensis BCRC 23118T (GU646863)
100
 R. taiwanensis RS1 (KC176700)
100
 R. minuta CBS 319T (AF189945)
 R. lactosa CBS 5826T (AF189936)
100
B
 R. marina CBS 2365T (AF189944)
100
 R. armeniaca CBS 8076T (AF189920)
 R. aurantiaca CBS 317T (AF189921)
100
 R. bacarum CBS 6526T (AF190002)
C
 R. hinnulea CBS 8079T (AF190003)
100
 R. phylloplana CBS 8073T (AF190004)
100
 Candida glabrata CBS 138T (AY497670)
Fig. S1.
0.02

## Slide 2
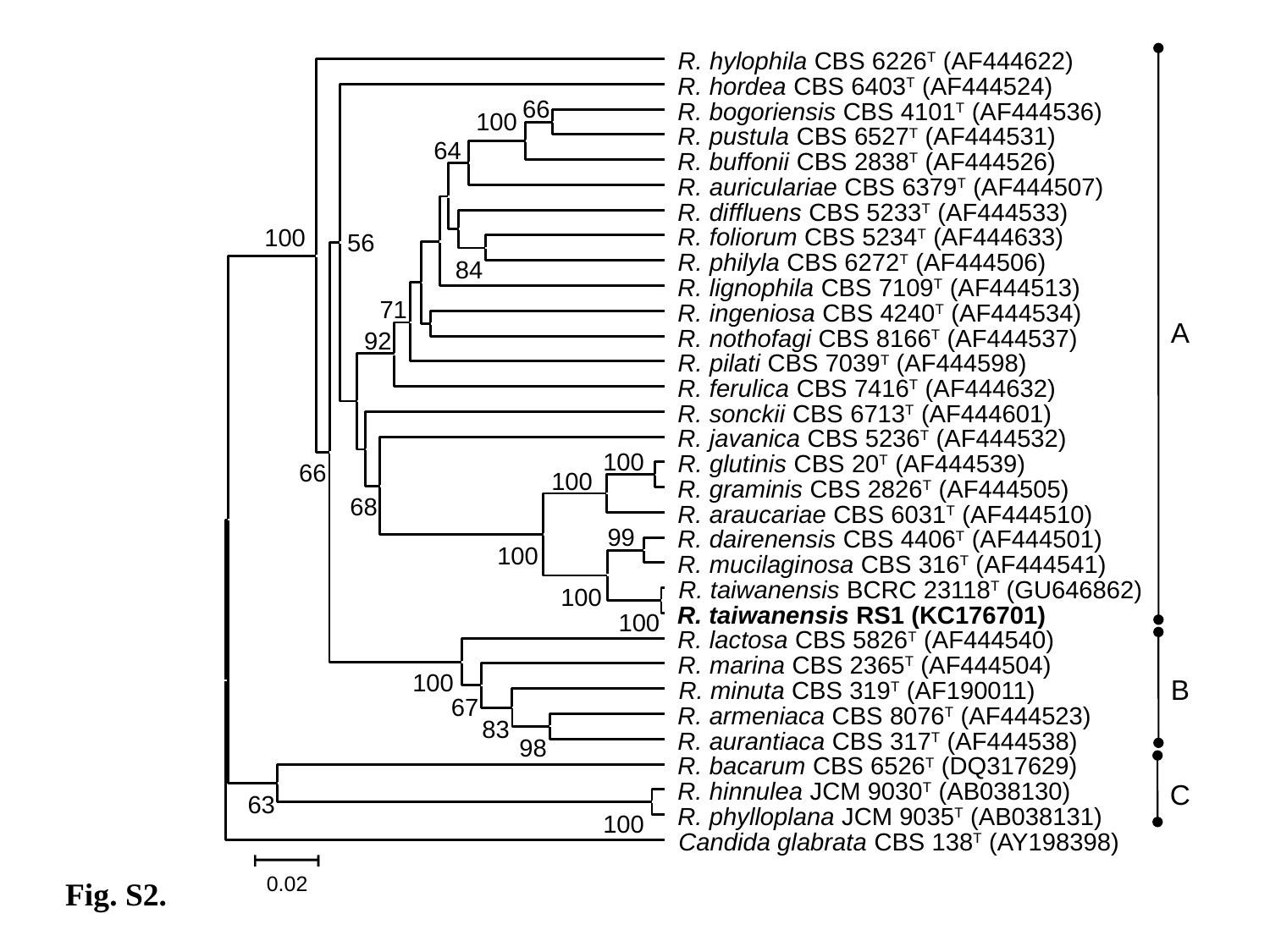

R. hylophila CBS 6226T (AF444622)
A
 R. hordea CBS 6403T (AF444524)
66
 R. bogoriensis CBS 4101T (AF444536)
100
 R. pustula CBS 6527T (AF444531)
64
 R. buffonii CBS 2838T (AF444526)
 R. auriculariae CBS 6379T (AF444507)
 R. diffluens CBS 5233T (AF444533)
 R. foliorum CBS 5234T (AF444633)
100
56
 R. philyla CBS 6272T (AF444506)
84
 R. lignophila CBS 7109T (AF444513)
71
 R. ingeniosa CBS 4240T (AF444534)
 R. nothofagi CBS 8166T (AF444537)
92
 R. pilati CBS 7039T (AF444598)
 R. ferulica CBS 7416T (AF444632)
 R. sonckii CBS 6713T (AF444601)
 R. javanica CBS 5236T (AF444532)
100
 R. glutinis CBS 20T (AF444539)
66
100
 R. graminis CBS 2826T (AF444505)
68
 R. araucariae CBS 6031T (AF444510)
99
 R. dairenensis CBS 4406T (AF444501)
100
 R. mucilaginosa CBS 316T (AF444541)
 R. taiwanensis BCRC 23118T (GU646862)
100
 R. taiwanensis RS1 (KC176701)
100
 R. lactosa CBS 5826T (AF444540)
B
 R. marina CBS 2365T (AF444504)
100
 R. minuta CBS 319T (AF190011)
67
 R. armeniaca CBS 8076T (AF444523)
83
 R. aurantiaca CBS 317T (AF444538)
98
 R. bacarum CBS 6526T (DQ317629)
C
 R. hinnulea JCM 9030T (AB038130)
63
 R. phylloplana JCM 9035T (AB038131)
100
 Candida glabrata CBS 138T (AY198398)
0.02
Fig. S2.

## Slide 3
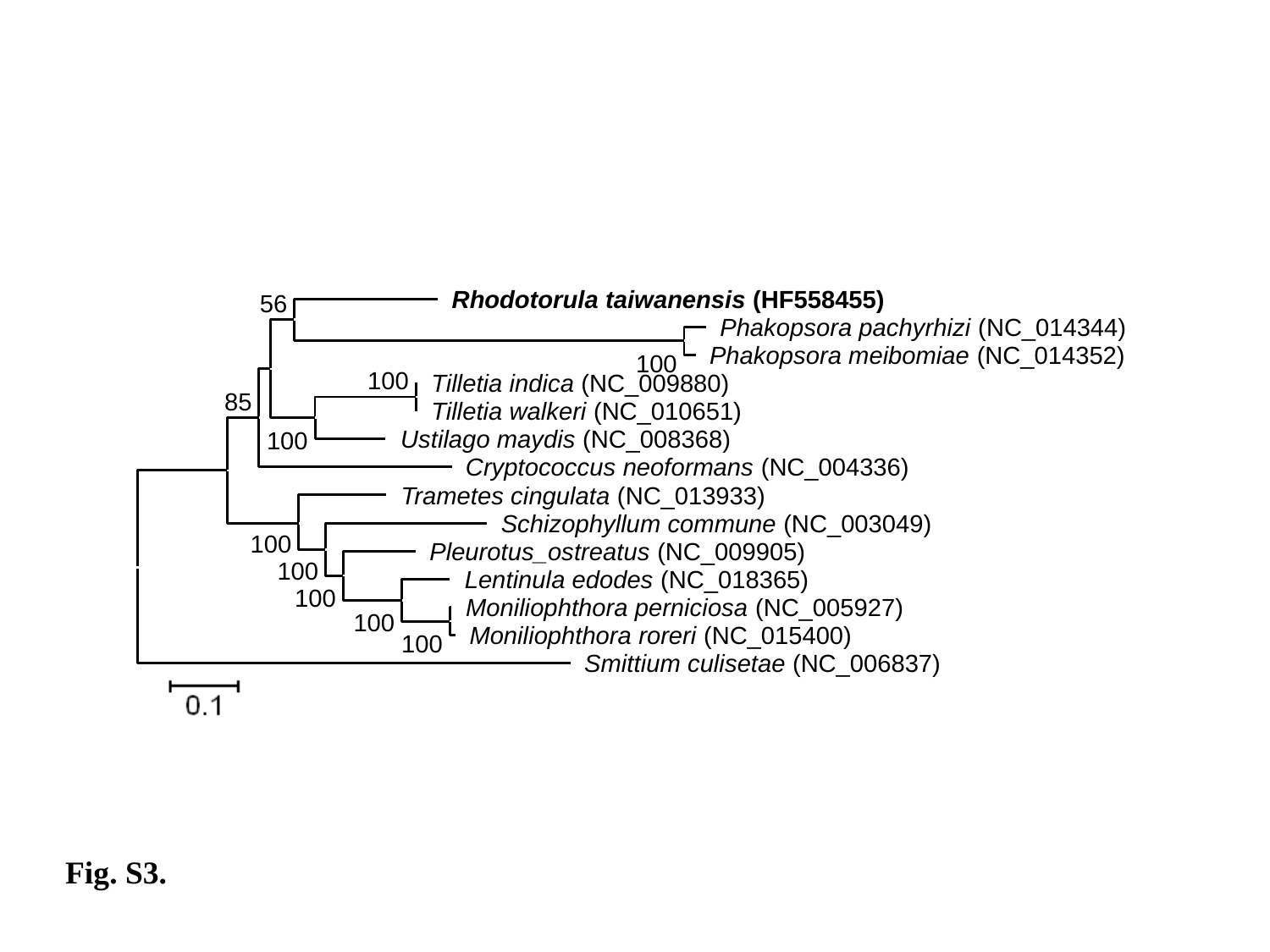

Rhodotorula taiwanensis (HF558455)
56
 Phakopsora pachyrhizi (NC_014344)
 Phakopsora meibomiae (NC_014352)
100
100
 Tilletia indica (NC_009880)
85
 Tilletia walkeri (NC_010651)
 Ustilago maydis (NC_008368)
100
 Cryptococcus neoformans (NC_004336)
 Trametes cingulata (NC_013933)
 Schizophyllum commune (NC_003049)
100
 Pleurotus_ostreatus (NC_009905)
100
 Lentinula edodes (NC_018365)
100
 Moniliophthora perniciosa (NC_005927)
100
 Moniliophthora roreri (NC_015400)
100
 Smittium culisetae (NC_006837)
Fig. S3.

## Slide 4
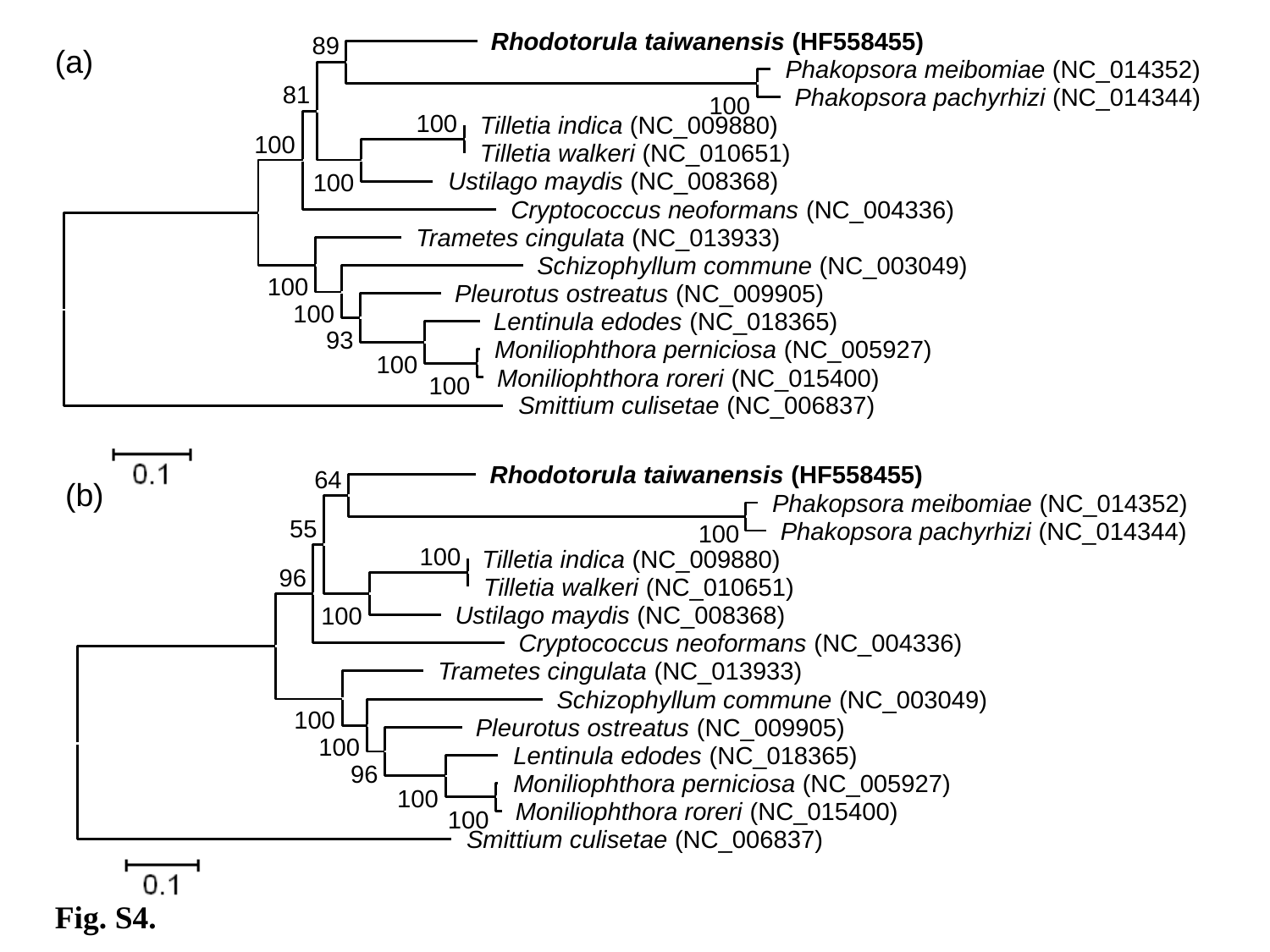

Rhodotorula taiwanensis (HF558455)
89
(a)
 Phakopsora meibomiae (NC_014352)
81
 Phakopsora pachyrhizi (NC_014344)
100
100
 Tilletia indica (NC_009880)
100
 Tilletia walkeri (NC_010651)
 Ustilago maydis (NC_008368)
100
 Cryptococcus neoformans (NC_004336)
 Trametes cingulata (NC_013933)
 Schizophyllum commune (NC_003049)
100
 Pleurotus ostreatus (NC_009905)
100
 Lentinula edodes (NC_018365)
93
 Moniliophthora perniciosa (NC_005927)
100
 Moniliophthora roreri (NC_015400)
100
 Smittium culisetae (NC_006837)
 Rhodotorula taiwanensis (HF558455)
64
(b)
 Phakopsora meibomiae (NC_014352)
55
 Phakopsora pachyrhizi (NC_014344)
100
100
 Tilletia indica (NC_009880)
96
 Tilletia walkeri (NC_010651)
 Ustilago maydis (NC_008368)
100
 Cryptococcus neoformans (NC_004336)
 Trametes cingulata (NC_013933)
 Schizophyllum commune (NC_003049)
100
 Pleurotus ostreatus (NC_009905)
100
 Lentinula edodes (NC_018365)
96
 Moniliophthora perniciosa (NC_005927)
100
 Moniliophthora roreri (NC_015400)
100
 Smittium culisetae (NC_006837)
Fig. S4.

## Slide 5
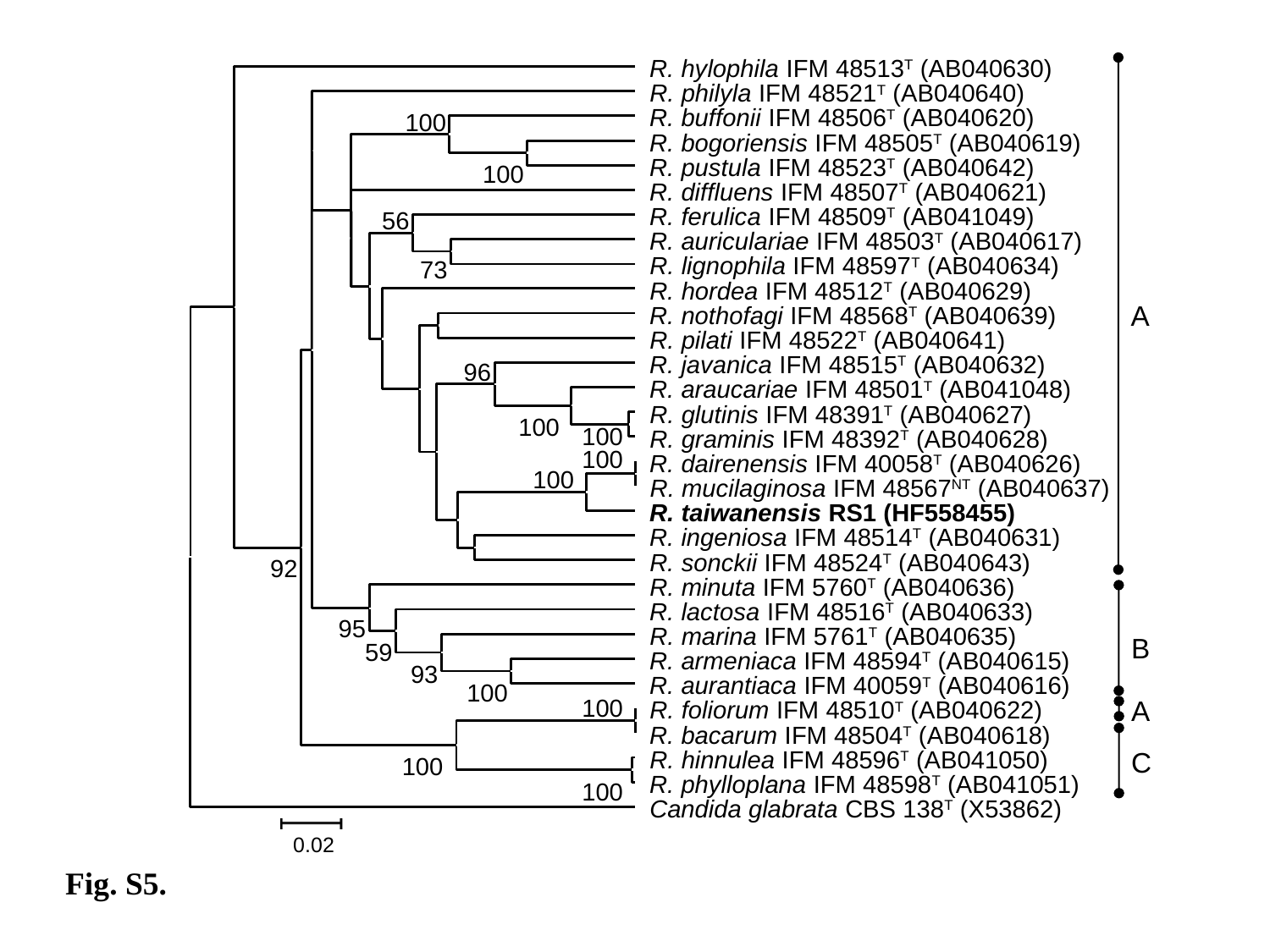

R. hylophila IFM 48513T (AB040630)
A
 R. philyla IFM 48521T (AB040640)
 R. buffonii IFM 48506T (AB040620)
100
 R. bogoriensis IFM 48505T (AB040619)
 R. pustula IFM 48523T (AB040642)
100
 R. diffluens IFM 48507T (AB040621)
 R. ferulica IFM 48509T (AB041049)
56
 R. auriculariae IFM 48503T (AB040617)
 R. lignophila IFM 48597T (AB040634)
73
 R. hordea IFM 48512T (AB040629)
 R. nothofagi IFM 48568T (AB040639)
 R. pilati IFM 48522T (AB040641)
 R. javanica IFM 48515T (AB040632)
96
 R. araucariae IFM 48501T (AB041048)
 R. glutinis IFM 48391T (AB040627)
100
100
 R. graminis IFM 48392T (AB040628)
100
 R. dairenensis IFM 40058T (AB040626)
100
 R. mucilaginosa IFM 48567NT (AB040637)
 R. taiwanensis RS1 (HF558455)
 R. ingeniosa IFM 48514T (AB040631)
 R. sonckii IFM 48524T (AB040643)
92
 R. minuta IFM 5760T (AB040636)
B
 R. lactosa IFM 48516T (AB040633)
95
 R. marina IFM 5761T (AB040635)
59
 R. armeniaca IFM 48594T (AB040615)
93
 R. aurantiaca IFM 40059T (AB040616)
100
A
100
 R. foliorum IFM 48510T (AB040622)
 R. bacarum IFM 48504T (AB040618)
C
 R. hinnulea IFM 48596T (AB041050)
100
 R. phylloplana IFM 48598T (AB041051)
100
 Candida glabrata CBS 138T (X53862)
0.02
Fig. S5.

## Slide 6
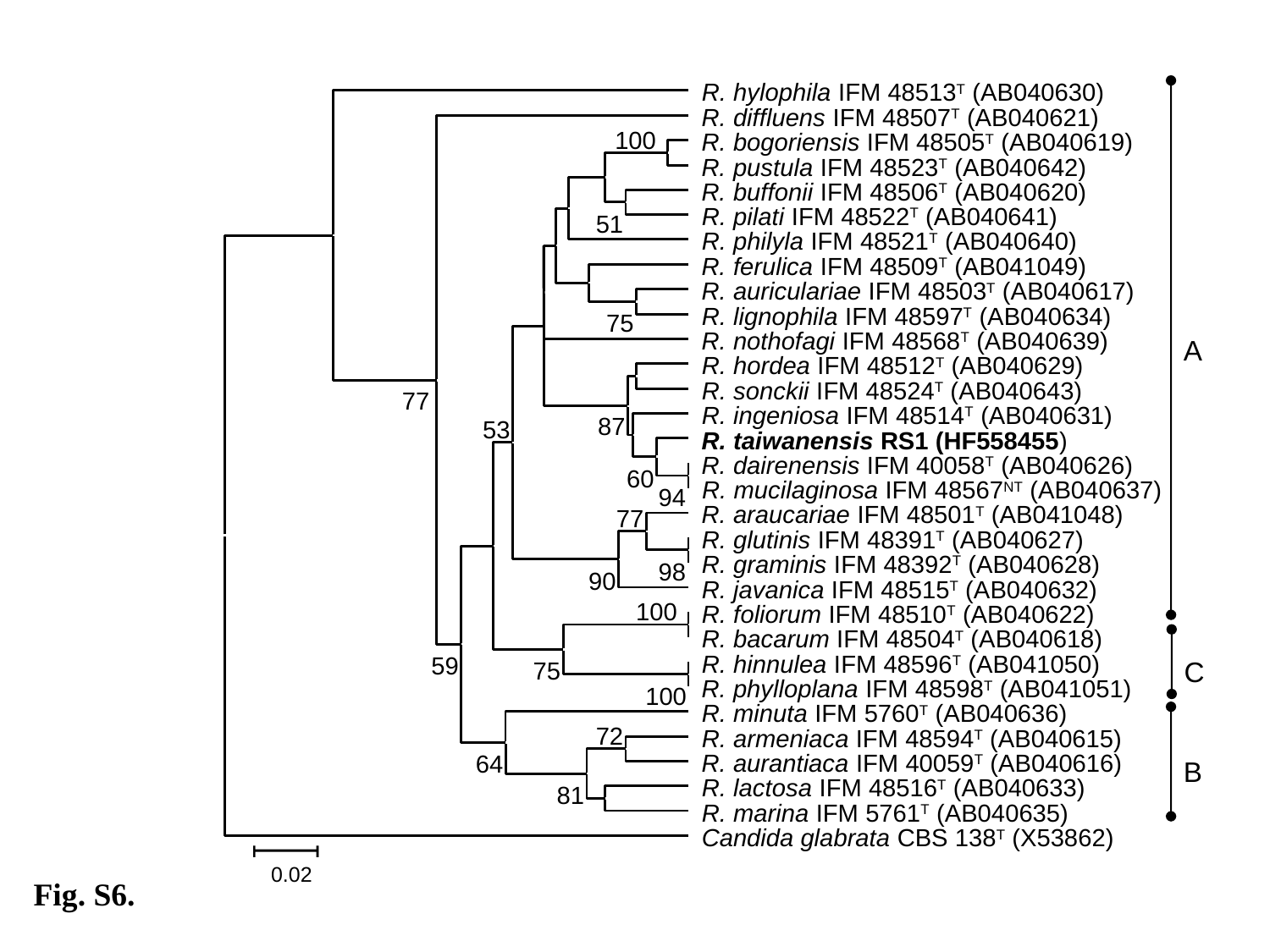

R. hylophila IFM 48513T (AB040630)
 R. diffluens IFM 48507T (AB040621)
100
 R. bogoriensis IFM 48505T (AB040619)
 R. pustula IFM 48523T (AB040642)
 R. buffonii IFM 48506T (AB040620)
 R. pilati IFM 48522T (AB040641)
51
 R. philyla IFM 48521T (AB040640)
 R. ferulica IFM 48509T (AB041049)
 R. auriculariae IFM 48503T (AB040617)
 R. lignophila IFM 48597T (AB040634)
75
 R. nothofagi IFM 48568T (AB040639)
A
 R. hordea IFM 48512T (AB040629)
 R. sonckii IFM 48524T (AB040643)
77
 R. ingeniosa IFM 48514T (AB040631)
87
53
 R. taiwanensis RS1 (HF558455)
 R. dairenensis IFM 40058T (AB040626)
60
 R. mucilaginosa IFM 48567NT (AB040637)
94
 R. araucariae IFM 48501T (AB041048)
77
 R. glutinis IFM 48391T (AB040627)
 R. graminis IFM 48392T (AB040628)
98
90
 R. javanica IFM 48515T (AB040632)
100
 R. foliorum IFM 48510T (AB040622)
 R. bacarum IFM 48504T (AB040618)
C
 R. hinnulea IFM 48596T (AB041050)
59
75
 R. phylloplana IFM 48598T (AB041051)
100
 R. minuta IFM 5760T (AB040636)
72
 R. armeniaca IFM 48594T (AB040615)
 R. aurantiaca IFM 40059T (AB040616)
B
64
 R. lactosa IFM 48516T (AB040633)
81
 R. marina IFM 5761T (AB040635)
 Candida glabrata CBS 138T (X53862)
0.02
Fig. S6.
